# Technofunctional and Sensory Properties of Fermented Lupin Protein Isolates

**DOI:** 10.3390/foods8120678

**Published:** 2019-12-13

**Authors:** Katharina Schlegel, Anika Leidigkeit, Peter Eisner, Ute Schweiggert-Weisz

**Affiliations:** 1 Chair of Aroma and Smell Research, Department of Chemistry and Pharmacy, Emil Fischer Center, Friedrich-Alexander-Universität Erlangen-Nürnberg, 91054 Erlangen, Germany; katharina.schlegel@ivv.fraunhofer.de; 2 Department Food Process Development, Fraunhofer Institute for Process Engineering and Packaging (IVV), 85354 Freising, Germany; anika.leidigkeit@ivv.fraunhofer.de (A.L.);; 3 ZIEL-Institute for Food & Health, TUM School of Life Sciences Weihenstephan, Technical University of Munich, 85354 Freising, Germany

**Keywords:** fermentation, lupin, plant protein, aroma profile, techno-functional properties, lactobacteria, foam, SDS-PAGE, solubility, emulsifying capacity

## Abstract

Lupin protein isolate was fermented with eight different microorganisms to evaluate the influence on sensory profile, techno-functional properties and protein integrity. All investigated microorganisms were able to grow in lupin protein isolate. The results showed that the foaming activity in the range of 1646–1703% and the emulsifying capacity in the range of 347–595 mL of the fermented lupin protein isolates were similar to those of the unfermented ones. Protein solubility at pH 4 showed no significant changes compared to unfermented lupin protein isolate, whereas the solubility at pH 7 decreased significantly from 63.59% for lupin protein isolate to solubilities lower than 42.35% for fermented lupin protein isolate. Fermentation with all microorganisms showed the tendency to decrease bitterness from 2.3 for lupin protein isolate (LPI) to 1.0–2.0 for the fermented ones. The most promising microorganisms for the improvement of the sensory properties of lupin protein isolates were *Lactobacillus brevis* as it reduced the intensity of characteristic aroma impression (*pea-like*, *green bell pepper-like*) from 4.5 to 1.0. The SDS-PAGE results showed the fermentation treatment appeared not to be sufficiently effective to destruct the protein integrity and thus, deplete the allergen potential of lupin proteins. Fermentation allows the development of food ingredients with good functional properties in foam formation and emulsifying capacity, with a well-balanced aroma and taste profile.

## 1. Introduction

The human organism relies on the nutritional intake of protein to maintain its health. The supply of animal protein has drastic environmental impacts on land use, air and water quality, and greenhouse gases [1]. The partial replacement of animal proteins by plant proteins could be a promising strategy to reduce the environmental impact of nutrition [2]. Soy protein is currently one of the most common plant proteins, but has some disadvantages including deforestation and genetic modification. Therefore, the search for alternative high-quality plant protein sources is a persistent challenge.

Lupins of the family *Fabaceae* are widely cultivated in Europe and are rich in seed proteins with valuable functional properties and a well-balanced amino acid profile [3,4]. However, like other leguminous plant proteins, lupin protein preparations exhibit a distinct aroma profile. Schlegel et al. [5] described the aroma profile of lupin protein isolate with oatmeal-like and fatty, cardboard-like impressions followed by earthy, moldy, beetroot-like; grassy; metallic; cooked potato-like and pea-like impressions. In addition, taste perceptions of bitter, salty, and astringent were identified in the taste pattern [5]. This characteristic aroma profile makes lupin proteins less suitable for some food applications as, for example, plain non-dairy based products. 

The lactic fermentation of legume proteins might be a promising approach to influence the sensory profile of these ingredients as various studies have shown that lactic fermentation of plant proteins results in a reduction or masking of off-flavors in legumes [6,7]. The influence of the fermentation on the aroma profile of the plant proteins strongly depends on the microorganisms used [6,7,8]. For the development of ingredients with specific aroma and taste properties (e.g., plant-based dairy alternatives or meat substitutes), different microorganisms must be investigated for their ability to improve the aroma profile of lupin proteins.

Besides the sensory profile, appropriate techno-functional properties of the proteins, such as solubility, emulsifying capacity and foam properties, are also a prerequisite for their use as food ingredients. Several authors have shown that the lactic fermentation of plant proteins affected their techno-functional properties in particular the protein solubility as shown by Lampart-Szczapa et al. [9] and Meinlschmidt et al. [10]. *Lactobacilli* produce metabolites such as organic acids, which alter the conformation of proteins and thus their physicochemical properties as relevant factors for their techno-functional behavior.

In addition, several studies have shown that the fermentation of plant proteins can reduce their allergenic potential [10,11] due to proteolytic enzyme activities. In particular for soy proteins, the impact of fermentation on the reduction of the allergenic potential has been investigated extensively [10,11,12,13,14]. It could be shown that the molecular weight distribution of the soy proteins could give a first indication of the degradation of the main allergens [10]. Data for lupins are still missing.

The objective of this study was to investigate the impact of lactic fermentation on the changes of the sensory profile of lupin protein isolates to improve the sensory properties of this promising food ingredient. Furthermore, the functional properties of the fermented isolates were also examined. To get a first indication of the reduction of the allergenic potential of the lupin proteins, the molecular weight distribution of the individually fermented protein samples were compared to the unfermented isolates.

## 2. Materials and Methods

### 2.1. Lupin Seeds

Lupin (*Lupinus angustifolius* cultivar Boregine) seeds were purchased from Saatzucht Steinach GmbH & Co KG (Steinach, Germany).

### 2.2. Preparation of Lupin Protein Isolate

Lupin protein isolate (LPI) was prepared from *Lupinus angustifolius* L. cultivar Boregine. Seeds were dehulled with an under runner disc sheller and separated and classified using an air-lift system. The dehulled seeds were passed through a counter-rotating roller mill and the resulting flakes were de-oiled in *n-*hexane. The processed flakes were suspended in 0.5 M HCL (pH 4.5) at a 1:8 (*w/w*) ratio and stirred for 1 h. After this, flakes were recovered in a decanter centrifuge with 5600× *g* at 4 °C for 1 h. Pre-extracted flakes were dispersed in 0.5 M NaOH (pH 8) at a 1:8 ratio for 1 h. After extraction, the suspension was centrifuged at 5600× *g* at 20 °C for 1 h and then neutralized (0.5 M NaOH), pasteurized (70 °C, 10 min), and spray dried.

### 2.3. Microbiological Compositions

#### 2.3.1. Nutrient Media

Liquid growth media and agar were purchased from Carl Roth (Karlsruhe, Germany) and Merck KGaA (Darmstadt, Germany).

#### 2.3.2. Bacteria Strains and Culture Conditions

Microorganisms were purchased from Deutsche Sammlung von Mikroorganismen und Zellkulturen (Braunschweig, Germany), Lehrstuhl für Technische Mikrobiologie, Technische Universität München (Freising, Germany) and Prof. Werner Back (Table 1). The microorganisms were stored as a cryo-culture in our strain collection and were recovered on MRS (De Man, Rogosa, & Sharpe) and TSYE (Trypticase soy yeast extract) agar. The selection of the microorganisms based on the results of previous experiments in which 26 microorganisms were tested for growth in LPI (data not shown). The microorganisms investigated in this study were the eight most promising microorganisms and were further processed.

Liquid cultures were incubated at aerobic (*Lactobacillus reuteri*, *Lactobacillus brevis*, *Lactobacillus amylolyticus*, *Lactobacillus sakei* subsp. *carnosus*, *Staphylococcus xylosus*, *Lactobacillus parabuchneri*) and anaerobic (*Lactobacillus helveticus*, *Lactobacillus delbrueckii*) conditions in sealed tubes (Sarstedt AG & Co, Nümbrecht, Germany) without shaking. Liquid pre-cultures (15 mL) were prepared from single colonies on agar plates and incubated for 36–48 h at 30 °C (*L. parabuchneri*, *L. brevis*), 37 °C (*L. helveticus*, *L. delbrueckii*, *L. sakei* subsp. *carnosus*, *L. reuteri*, *S. xylosus*) and 42 °C (*L. amylolyticus*), respectively.

#### 2.3.3. Determining Growth Conditions of Microorganisms

Growth curves were recorded with a microplate reader (Infinite M1000 Pro, Tecan Group Ltd., Männedorf, Switzerland) measuring with an OD of 600 nm each 15 min with previous shaking at various temperatures. Aliquots of 200 µL liquid medium in 96 well micro test plates were inoculated from pre-cultures. For anaerobic conditions, cultures were covered with 100 µL sterile paraffin oil. 

#### 2.3.4. Determination of pH and Viable Cell Counts

The pH course during lupin fermentation was recorded for 24 h with one measurement point each 30 min with a wtw pH 3310 pH electrode (Xylem Analytics Germany GmbH, Weilheim, Germany). The viable cell counts were determined on nutrients agar from 1 mL of diluted (0.9% NaOH) culture aliquots and expressed as log_10_ of colony forming units per milliliter per sample (CFU/mL).

### 2.4. Fermentation of Lupin Protein Isolates

Fermentation of LPI was carried out in a 5 L glass reaction vessel in an incubator under aerobic conditions and in a 5 L glass reaction vessel with a fermenter (Satorius) under anaerobic conditions, respectively. A 5% LPI (*w/w*) solution with 0.5% glucose (*w/w*) was pasteurized separately at 80 °C for 20 min and mixed under sterile conditions. LPI solution was inoculated with the activated culture in the late exponential growth phase (10^7^ CFU/mL). Anaerobic conditions were achieved by flushing the reactor with N_2_. The inoculated lupin protein isolate was incubated for 24 h without stirring and sampled at 0, 4, 18, and 24 h. Fermentation was stopped by heat treatment at 90 °C for 20 min. All samples were neutralized (pH 7) with 1 M NaOH and spray-dried with a Niro Atomizer 2238 (GEA, Düsseldorf, Germany). The whole experiment was repeated in duplicate.

### 2.5. Analysis of d-Glucose

For determination of ᴅ-glucose, 100 µL of the sample was mixed with 450 µL zinc sulphate (10%) and 450 µL NaOH (0.5 M) and incubated for 20 min at room temperature. After incubation, the sample was centrifuged at 12,045× *g* for 10 min and the supernatant was filtrated using a 0.45 µm nylon filter. d-Glucose was analyzed using the enzymatic d-glucose test from R-Biopharm (Darmstadt, Germany) following the manufacturer’s instructions.

### 2.6. Chemical Composition

The protein content was estimated based on the nitrogen content according to the Dumas combustion method (AOAC 968.06) with a factor of N × 5.8 [15] using a Nitrogen Analyzer FP 528 (Leco Corporation, St. Joseph, MI, USA). The dry matter was identified according to AOAC methods 925.10 in a TGA 601 thermogravimetric system (Leco Corporation) at 105 °C.

### 2.7. Sensory Analysis

#### 2.7.1. Panelists

The panel consisted of trained volunteers recruited from Fraunhofer IVV (Freising, Germany), exhibiting no known illness at the time of examination and with normal olfactory function. The panel consisted of 10 panelists (eight female and two male). All panelists were trained in weekly training sessions with selected, super-threshold aroma solutions to correctly identify and name fragrances.

#### 2.7.2. Sensory Evaluation

For sample evaluation, 2% (*w/w*) solutions of the LPI and fermented LPI, respectively, and tap water were prepared by stirring. The samples were evaluated in two sessions on one day. Each panelist received five samples of 20 mL aliquots (room temperature) in the first session and four samples in the second session in covered glass vessels, with tap water and flavorless crackers used to neutralize between each sample. To obtain the retronasal aroma and taste attributes, panelists were required to open the lid of the beaker and record the retronasal aroma and taste attributes. After the discussion regarding the most prominent aroma and taste attributes, panelists were asked to select and rate attributes on a scale from 0 (no perception) to 10 (strong perception) that were perceived by at least half of the panel. In addition, the panelists were asked to rate the overall intensity of the aroma of the sample also on a scale from 0 (no perception) to 10 (strong perception), and a hedonic scaling was performed on a scale from 0 (strong dislike) to 5 (neutral) to 10 (strong like). We used supra-threshold aroma solutions for orthonasal perception as a reference for each selected aroma attribute.

### 2.8. Techno-Functional Properties

#### 2.8.1. Protein Solubility

The solubility (%) of LPI and fermented LPI was measured in duplicate at pH 4 and 9 according to Morr et al. [16]. For each measurement, 1.5 g of protein was suspended in 50 mL 0.1 M NaCl and the pH was adjusted with 0.1 M NaOH or 0.1 M HCl. After stirring for 1 h at room temperature, the non-dissolved fractions of the samples were separated by centrifugation (20,000× *g*, 15 min, room temperature) and the supernatants were passed through Whatman No. 1 filter paper to remove any remaining particulates. The protein content of the supernatant was determined by Lowry et al. [17] using the DC Protein Assay (Bio-Rad Laboratories, Hercules, CA, USA). The absorbance was read at 750 nm and the protein concentration was calculated using the generated BSA standard curve. The resulting amount of dissolved proteins was related to the total amount of protein and the protein solubility (%) was determined.

#### 2.8.2. Foam Properties

For the determination of the foaming activity, 50 mL of a 5% (*w/w*) protein solution (pH 7) was whipped at room temperature for 8 min in a Hobart 50-N device (Hobart GmbH, Offenburg, Germany) according to the method described by Phillips et al. [18]. The increase of foam volume was used to determine the foam activity. The percentage leftover of foam volume after 1 h was defined as the foaming stability (%).

#### 2.8.3. Emulsifying Capacity

Emulsifying capacity (EC) was identified according to Wang and Johnson [19]. Samples were dispersed in deionized water (1%, *w/w*), adjusted to pH 7 and stirred with an Ultraturrax at 18 °C. Rapeseed oil was continuously added using a Titrino 702 SM titration system (Metrohm GmbH & Co. KG, Hertisau, Switzerland) at a rate of 10 mL/min until phase inversion was detected by means of an LF 521 meter fitted with a KLE1/T electrode (Wissenschaftlich-technische Werkstätten GmbH, Weilheim, Germany). For EC calculation, volume of oil needed to achieve the phase inversion was used (mL oil per g sample). Measurement was repeated in duplicate.

### 2.9. Analysis of the Molecular Weight Profiles of LPI and Fermented LPI

The molecular weight distribution of the untreated and fermented LPI was determined by sodium dodecyl sulfate-polyacrylamide gel electrophoresis (SDS-PAGE) as described by Laemmli [20] with modification under reducing conditions. Untreated LPI, fermented LPI and control samples were re-suspended in 1 mL loading buffer (0.125 mol/l Tris-HCl, 4% SDS (*w/v*), 20% glycerol (*v/v*), 0.2 mol/l DDT, 0.02% bromophenol blue, pH 6.8), dissolved for 30 min at 30 °C in an ultrasonic bath and boiled for 5 min at 95 °C in an Eppendorf ThermoMixer C (Eppendorf AG, Hamburg, Germany). Following centrifugation at 12,045× *g* for 10 min (Mini Spin, Eppendorf AG), an aliquot of the supernatant was transferred to a fresh tube and supplemented in a ratio of 1:10 with loading buffer (see above). An aliquot of 10 µL of each sample was transferred into the wells of pre-cast Criterion TGX stain-free 12% polyacrylamide gels (Bio-Rad Laboratories, Hercules, CA, USA). The samples were separated for 36 min at 200 V (60 mA, 100 W) (Amersham Biosciences Europe GmbH, Freiburg, Germany) at room temperature in a vertical electrophoresis cell (Bio-Rad Laboratories) with a 10–250 kDa Precision Plus Protein Unstained Standard (Bio-Rad Laboratories) alongside as size markers. Protein subunits were visualized using a Gel Doc™ EZ Imager system (Bio-Rad Laboratories). The molecular weight distribution was determined using Image Lab software (Bio-Rad Laboratories).

### 2.10. Statistical Analysis

Results are expressed as means ± standard deviations and for sensory evaluation (aroma profile) as median ± standard deviations. Data were analyzed using pairwise *t*-test to determine the significance of differences between a sample and the unfermented LPI, with a threshold of *p* < 0.05. For the microbial growth (CFU, pH, glucose), data were analyzed using one-way analysis of variances (ANOVA) and means were generated and adjusted with Tukey’s honestly significant difference post hoc test to determine the significance of differences between all samples, with a threshold of *p* < 0.05. Statistical analysis was performed with SigmaPlot 12.5 for Windows (Systat Software GmbH, Erkrath, Germany).

## 3. Results and Discussion

### 3.1. Chemical Composition

Dry matter and protein content of LPI and fermented LPI are given in Table 2. 

Dry matter of LPI and fermented LPI ranged from 92.8% for *L. delbrueckii* to 95.4% for unfermented LPI. Unfermented LPI contained the highest protein content with 89.6%.

### 3.2. Comparison of Microbial Growth on Lupin Protein Isolate Solutions

**Figure 1:**
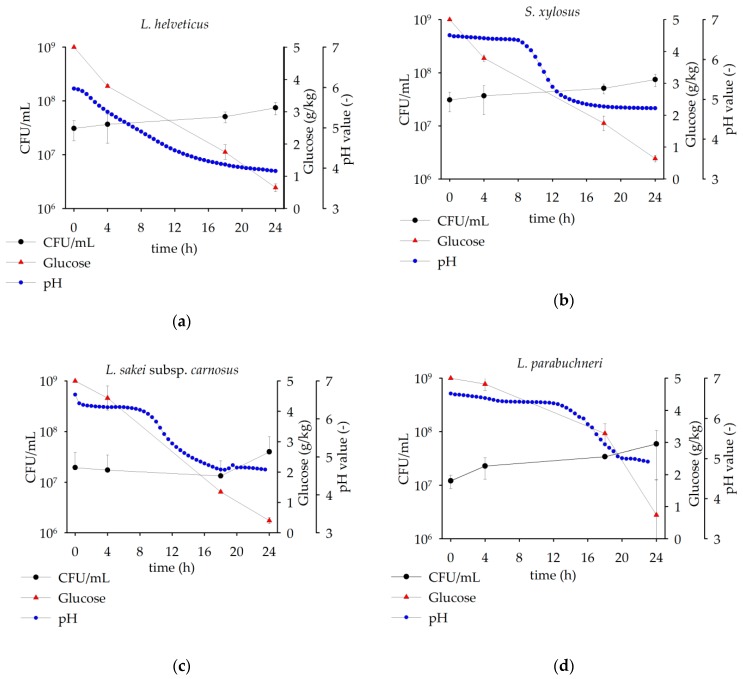
CFU/mL, glucose amount, and pH after 0 h, 4 h, 18 h, and 24 h for *L. helveticus* (**a**), *S. xylosus* (**b**), *L. sakei* subsp. *carnosus* (**c**), *and L. parabuchneri* (**d**). The data are expressed as mean ± standard deviation from duplicates.

The growing parameters consisting of CFU, pH and glucose for all eight microorganisms investigated are shown in Table 3. In addition, growth curves of four of these microorganisms were selected in Figure 1 in order to highlight the temporally different transitions into the exponential phase. 

The results showed that all microorganisms were able to grow in LPI solution. The minimum increase in CFU/mL (ΔE_CFU_) was recorded for *L. reuteri* with 1.36×10^7^ CFU/mL and the maximum for *S. xylosus* with 6.01×10^8^ CFU/mL. The results of the pH curve showed that *L. amylolyticus* and *L. helveticus* appear to have the best metabolism and were adapted most rapidly to the lupine solution. The pH curves showed a direct and constant pH acidification over 24 h for *L. helveticus* (exemplarily shown in Figure 1a) and *L. amylolyticus* (similar to Figure 1a). After 24 h of fermentation, a change into the stationary phase could not be observed for both microorganisms. *S. xylosus* (exemplarily shown in Figure 1b) and *L. delbrueckii* (similar to Figure 1b) showed a lag phase and a transition into the log phase after approximately 8 h. *S. xylosus* reached the stationary phase after 14 h. *L. delbrueckii* did not show a clear transition into the stationary phase after 24 h. *L. sakei* subsp. *carnosus* (exemplarily shown in Figure 1c) and *L. reuteri* (similar to Figure 1c) changed from the lag phase to the log phase after 10 h and reached the stationary phase after 18 h. The largest lag phases were recorded for *L. parabuchneri* (exemplarily shown in Figure 1d) and *L. brevis* (similar to Figure 1d). Both microorganisms reached the log phase after 14 h and changed to the stationary phase after 20 h. The results of glucose concentrations showed that the added carbon source of 5 g/kg glucose was metabolized by all microorganisms. After 24 h fermentation, residues of glucose were present in all fermented samples. The degradation of glucose (ΔE_Glucose_) ranged from 3.4 g/kg with remaining 1.7 g/kg glucose after 24 h for *L. amylolyticus* to 4.8 g/kg with remaining 0.2 g/kg glucose after 24 h for *L. reuteri*, respectively. Fritsch et al. [21] and Lampart-Szczapa, Konieczny, Nogala-Kałucka, Walczak, Kossowska and Malinowska [9] showed similar results and confirmed the suitability of lupine flour and lupine protein, respectively, for lactic fermentation.

### 3.3. Sensory Anaylsis

In the retronasal sensory evaluation, the following six aroma qualities and corresponding references were selected by the panelists for the description of LPI and fermented LPI: *cheesy* (butanoic acid); *popcorn-like*, *roasty* (2-acetylpyrazine); *earthy*, *moldy*, *beetroot-like* (geosmin); *pea-like*, *green bell pepper-like* (2-isopropyl-3-methoxypyrazine); *cooked potato-like* (3-(methylthio-)propanal); and *oatmeal-like*, *fatty* (oatmeal).

**Figure 2:**
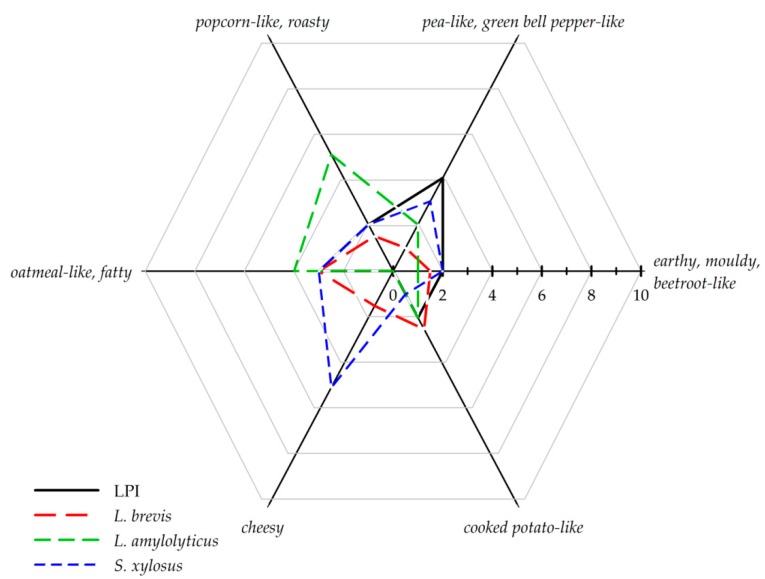
Retronasal aroma profile analyses of LPI, *L. brevis*, *L. amylolyticus*, and *S. xylosus* fermented samples on a scale from (no perception) to 10 (strong perception). The data are displayed as median values of the sensory evaluations (*n* = 10).

Comparative aroma profile analyses (Figure 2) of LPI, *L. brevis*, *L. amylolyticus* and *S. xylosus* were emphasized to highlight the increase of aroma perception in *cheesy* and *roasty*, *popcorn-like* as well as the reduction of the mean aroma perceptions in comparison to LPI, across the panel. The primary aroma attributes in the LPI were *pea-like*, *green bell pepper-like*, and *oatmeal-like*, *fatty* with median values of 4.5 and 3.0, respectively. Otherwise, the aroma impression was evaluated with low intensities for *earthy*, *moldy*, *beetroot-like*; *cooked potato-like*, and *popcorn-like*, *roasty* with median values of 2.5, 2.5, and 1.0, respectively, while the attribute *cheesy* was imperceptible (median value of 0). The dominant aroma attributes in the LPI samples obtained after fermentation with *L. reuteri* were described as *cheesy* (median of 4.0) and *oatmeal-like*, *fatty* (median value of 3.0). The aroma perception was otherwise evaluated with low intensities for *earthy*, *moldy*, *beetroot-like*; *pea-like*, *green bell pepper-like*, and *cooked potato-like* with values of 2.0 and *popcorn-like*, *roasty* with a value of 1.0. The aroma perception of the samples obtained after *L. brevis* fermentation was evaluated with a maximum intensity of 3.0 for *oatmeal-like*, *fatty*. Followed by low intensities of *earthy*, *moldy*, *beetroot-like*; *cooked potato-like*, and *popcorn-like*, *roasty* with values of 2.0 and *cheesy* and *pea-like*, *green bell pepper-like* with values of 1.0. Fermentation of LPI with *L. amylolyticus* was described with a dominant aroma impression of *popcorn-like*, *roasty* with a value of 5.0, followed by *oatmeal-like*, *fatty* with a value of 3.5. Low intensities were judged for *pea-like*, *green bell pepper-like* with a value of 2.0, *cooked potato-like* with a value of 1.5, and *earthy*, *moldy*, *beetroot-like* with 1.0. Attribute *cheesy* was not perceptible (median value of 0). The aroma profile of the *L. parabuchneri* fermented samples showed the aroma impressions of *pea-like*, *green bell pepper-like*; *cheesy;* and *oatmeal-like*, *fatty* with intensities of 4.0, 3.0, and 3.0 respectively. Less dominant were the attributes *popcorn-like*, *roasty* and *cooked potato-like*, both with median values of 2.0, and *earthy*, *moldy*, *beetroot-like* with an intensity median value of 1.5. *L. sakei* subsp. *carnosus* fermentation was described with *popcorn-like*, *roasty* as main aroma impression with an intensity of 4.0, followed by *oatmeal-like*, *fatty* with an intensity of 3.0. The attributes *earthy*, *moldy*, *beetroot-like*; *pea-like*, *green bell pepper-like*, and *cooked potato-like* were described equally less intensely with values of 2.0. The attribute *cheesy* was imperceptible in the samples obtained after fermentation with *L. sakei* subsp. *carnosus.* Samples fermented with *S. xylosu*s exhibited a *cheesy* intensity of 5.0, followed by *oatmeal-like*, *fatty* with a value of 3.0. The other attributes were less intense with *pea-like*, *green bell pepper-like* (2.5), *earthy*, *moldy*, *beetroot-like* (2.0), *popcorn-like*, *roasty* (2.0), and *cooked potato-like* (1.0). Similar to *L. amylolyticus* and *L. sakei* subsp. *Carnosus* fermentation, the attribute *popcorn-like*, *roasty* was described as a dominant aroma impression for the *L. helveticus* fermented samples with an intensity of 4.0. The aroma profile of *L. helveticus* was otherwise described with aroma impressions *pea-like*, *green bell pepper-like* with a value of 3.0. The attributes *oatmeal-like*, *fatty*; *cooked potato-like*; *earthy*, *moldy*, *beetroot-like*, and *cheesy* were rated less intensively with values of 2.5, 2.0, 1.0, and 0.5 respectively. The main aroma impression of *L. delbrueckii* was assessed as *oatmeal-like*, *fatty* with an intensity of 4.0. The aroma impressions *popcorn-like*, *roasty*; *earthy*, *moldy*, *beetroot-like*; *pea-like*, *green bell pepper-like;* and *cooked potato-like* were rated with an intensity of 2.0. The attribute *cheesy* could not be observed by the panelists.

The aroma profile showed, with the exception of the *L. parabuchneri* fermented samples, that the aroma perception *pea-like*, *green bell pepper-like* decreased in intensity due to the fermentation. The maximum decreases were determined for the samples obtained after *L. brevis* fermentation with an intensity of 1.0 compared to unfermented LPI with a value of 4.5. Furthermore, fermentation increased the intensity of the aroma impressions *popcorn-like*, *roasty* and *cheesy*. In the samples fermented with *L. amylolyticus*, *L. sakei* subsp. *carnosus* and *L. helveticus* the aroma impressions *popcorn-like*, *roasty* increased from 1.0 for unfermented LPI to 5.0, 4.0 and 4.0 respectively. *S. xylosus* and *L. reuteri* showed an increase in intensity of the attribute *cheesy* from 0 for unfermented LPI to 5.5 and 4.0 respectively. Several authors confirm for lupin, soy and pea protein, respectively, the significant modification of aroma profile by fermentation [6,7,8,10]. The authors described the reduction of *n*-hexanal content, which contributes most to the *green* and *beany* off-flavor of pea, by fermentation with lactic acid extract in pea protein extract and soy, respectively [6,7,8]. Further studies showed that the fermentation of soy protein isolate with lactic acid bacteria significantly reduced the aroma impression of *beany* [10]. A statement about the reduction of *n*-hexanal content due fermentation cannot be obtained in this study. A reduced perception of the *pea-like*, *green bell pepper-like* aroma may also be caused by masking effects. The total aroma intensities of all samples differed only slightly with median values of 5.5 for the samples fermented with *L. amylolyticus*, 5.0 for LPI, *L. parabuchneri*, *S. xylosus*, *L. reuteri*, and *L. sakei* subsp. *carnosus* fermented samples, 4.5 for *L. helveticus* and *L. delbrueckii*, and 4.0 for *L. brevis* fermented samples.

**Figure 3:**
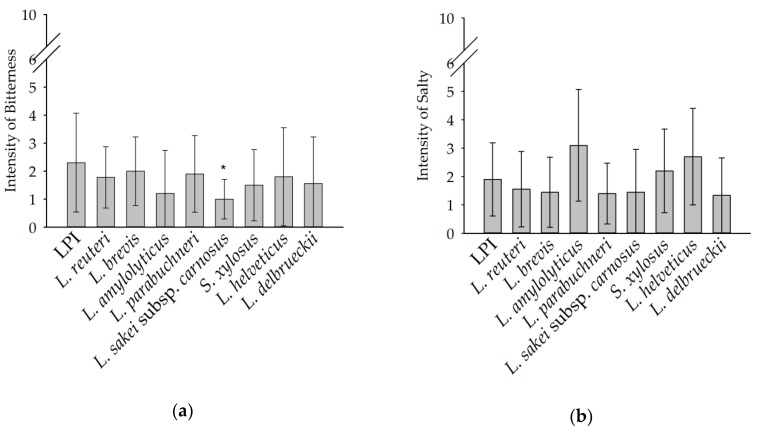
Intensity of bitter (**a**) and salty (**b**) taste perception of LPI and fermented LPI rate on a scale from 0 (no perception) to 10 (strong perception). Means marked with an asterisk (*) indicate significant differences between the individual sample and the unfermented LPI (*p* < 0.05) following pairwise *t*-test.

The taste impressions *bitter* and *salty* were analyzed by the panel in the sensory evaluation and displayed in Figure 3. The *bitter* intensity of unfermented LPI was described with a mean value of 2.3. All fermented samples did not differ significantly (*p* < 0.05) in bitterness compared to LPI, with the exception of *L. sakei* subsp. *carnosus* (1.0). However, the trend of the mean values of the fermented samples showed slightly lower intensities of bitterness compared to LPI. The intensity of *salty* was described for LPI with a mean value of 1.9. In comparison, no significant differences (*p* < 0.05) in the intensity of the fermented samples were found. However, the samples obtained after fermentation with *L. amylolyticus*, *S. xylosus*, and *L helveticus* tended to be more salty.

**Figure 4:**
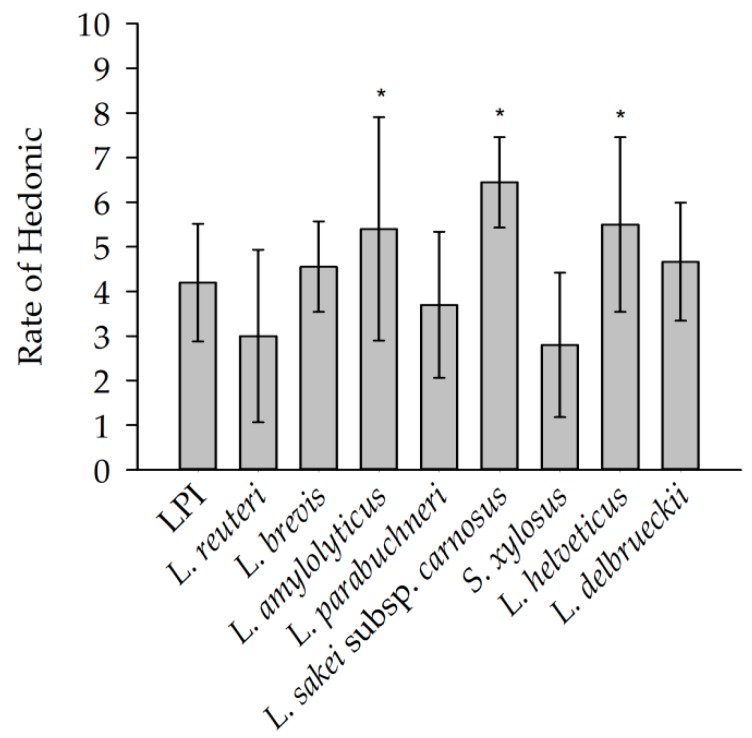
Rate of hedonic evaluation of LPI and unfermented LPI; scale from 0 (strong dislike) to 5 (neutral) to 10 (strong like). Means marked with an asterisk (*) indicate significant differences between the individual sample and the unfermented LPI (*p* < 0.05) following pairwise *t*-test.

The hedonic evaluation was performed for a first indication of the prevalence of the samples and was not performed according to ISO standards. The evaluation (Figure 4) of the panel resulted in a rating of 4.2 for unfermented LPI. The sample, which was most popular with the panelists (6.4), was fermented with *L. sakei* subsp. *carnosus*, followed by the samples fermented with *L. helveticus* (5.5) and *L. amylolyticus* (5.4). The most unpopular samples were the ones fermented with *S. xylosus* and *L. reuteri* with values of 2.8 and 3.0, respectively. The results showed that both saltiness and bitterness do not have a considerable effect on the acceptance of the samples. The acceptance seems to be influenced by the differences in the aroma profile. It was found that the samples with the aroma attributes popcorn like were rated as popular by all subjects. The samples with the maximum evaluation had the attribute *popcorn-like*, *roasty* in their aroma profile as dominant aroma impression and also the highest intensity in this attribute compared to LPI and all other fermented samples. Meanwhile, the aroma impression *cheesy* dominated in *S. xylosus* and *L. reuteri* fermented samples—the samples with the minimal hedonic rating. In addition, both of these samples had the highest intensity of this attribute compared to LPI and the other samples.

### 3.4. Techno-Functional Properties

#### 3.4.1. Protein Solubility

The protein solubility of LPI and fermented LPI was determined at pH 4 and pH 7 and given in Table 4. The protein solubility of all samples was higher under neutral condition (pH 7) than under acidic condition (pH 4). Usually, protein solubility is minimal at the isoelectric point (pH 4.5) [4,22,23,24]. Unfermented LPI showed a significantly (*p* < 0.05) higher protein solubility of 63.6% at pH 7 than all fermented samples. The minimum solubility was measured for the samples obtained after *L. helveticus* and *L. delbrueckii* fermentation with 23.57% and 27.48%, respectively, and the maximum solubility of the fermented samples with *L. reuteri* with 42.35%. Similar results have been found in other studies. Lampart-Szczapa, Konieczny, Nogala-Kałucka, Walczak, Kossowska, and Malinowska [9] determined also lower solubility of lupin proteins after fermentation than non-fermented samples and other authors observed this for fermented soy [10,11,25,26]. *Lactobacilli* produce organic acids during fermentation, which might have induced an irreversible coagulation of proteins and thus a reduced solubility. Further, heat treatment for the fermentation stop (90 °C, 20 min) might have promoted aggregation and cross-linking of partially hydrolyzed lupin proteins [10]. In contrast, the protein solubility at pH 4 after fermentation was not significantly different (*p* < 0.05) to unfermented LPI (7.31%), with the exception of the samples fermented with *L. delbrueckii* (5.55%) and *L. helveticus* (5.92%). The highest protein solubility at pH 4 was measured for *S. xylosus* fermented samples with 8.11%.

#### 3.4.2. Foam Properties

The foam properties (foam activity and stability) of LPI and fermented LPI are shown in Table 5. All fermented samples showed a significant higher (*p* < 0.05) foam activity compared to unfermented LPI, with the exception of the samples fermented with *L. brevis* and *L. delbrueckii*. *L. brevis* and *L. delbrueckii* fermented samples showed an increase in foam activity compared to unfermented LPI, although the differences were not significant (*p* < 0.05). Similar results were obtained by Klupsaite et al. [27] for lupin proteins and Meinlschmidt, Ueberham, Lehmann, Schweiggert-Weisz, and Eisner [10] for soy protein isolates.

Unfermented LPI showed a foam stability of 89%. The foam stability of all fermented samples, with exception of *L. parabuchneri* and *L. helveticus*, was above 80%. *L. parabuchneri* and *L. helveticus* showed significantly (*p* < 0.05) lower stability of 16% and 20%, respectively, compare to unfermented LPI.

#### 3.4.3. Emulsifying Capacity

Unfermented LPI showed an emulsifying capacity of 552.9 mg/mL (Table 6). The treatments with the various microorganisms resulted in emulsifying capacities in the range of 347.7 mg/mL for *L. parabuchneri* up to 595.6 mg/mL for *S. xylosus.* Samples fermented with *L. parabuchneri* (347.7 mg/mL), *L. delbrueckii* (370.3 mg/mL), *L. sakei* subsp. *carnosus* (407 mg/mL) and *L. brevis* (447 mg/mL), showed a significantly (*p* < 0.05) lower emulsifying capacity than the unfermented LPI sample. The residual samples did not show a significantly (*p* < 0.05) lower emulsifying capacity compared to unfermented LPI, but a tendency was observed. El-Adawy et al. [28] and Qi et al. [29] described a positive correlation between the emulsifying capacity and protein solubility of a protein. A reduced protein solubility was observed in fermented LPI compared to unfermented LPI. It could be an indication of the tendency decrease in emulsifying capacity of fermented LPI. The literature offers controversial data regarding the emulsifying capacity after fermentation. Lampart-Szczapa, Konieczny, Nogala-Kałucka, Walczak, Kossowska, and Malinowska [9] observed a decrease in emulsifying capacity after the fermentation of lupine protein while Klupsaite, Juodeikiene, Zadeike, Bartkiene, Maknickiene, and Liutkute [27] reported a significant increase in capacity after 24 h of fermentation. The fermentation of soy protein isolate resulted in a decrease in emulsifying capacity [10].

### 3.5. Analysis of the SDS-PAGE Profiles of Unfermented and Fermented (24 h) LPI

**Figure 5:**
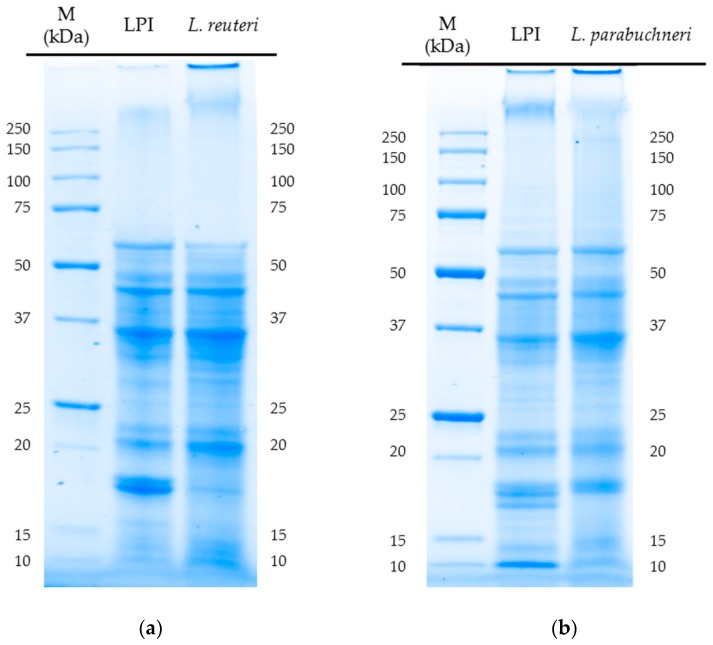
Peptide band profiles of fermented LPI produced by fermentation (24 h) with *L. reuteri* (**a**) and *L. parabuchneri* (**b**) as determined by SDS-PAGE under reducing conditions.

The molecular weight distribution of LPI and fermented LPI was analyzed to obtain an indication of the integrity of the proteins. The results of the SDS-PAGE showed that the microorganisms were able to change the SDS-PAGE profile of the lupin protein isolates. It could be observed that the polypeptides of lupin protein isolates were partially decomposed. All fermented samples showed a degradation of low molecular weight fractions (<23 kDa) associated with the increase in small polypeptides with a molecular weight of <15 kDa compared to unfermented LPI (data not presented). In particular, polypeptides with a molecular weight of 17 kDa appear to be decomposed in all samples and exemplarily shown for *L. reuteri* and *L. parabuchneri* in Figure 5. The amount of polypeptides within the medium molecular weight range (25–36 kDa) increased due to the breakage of larger polypeptides. It appears that all used microorganisms are proteolytic active to decompose medium molecular weight and low molecular weight polypeptides from lupin protein isolate into small fragments.

Meinlschmidt, Ueberham, Lehmann, Schweiggert-Weisz, and Eisner [10] also investigated the effect of fermentation with *L. helveticus* on the integrity of proteins in soy protein isolate. The number of subunits in the 25–37 kDa range was also increased, as were the number of low molecular weight bands <20 kDa.

## 4. Conclusions

Fermentation maintains the functional properties and improves the sensory properties of lupin protein isolate. Following the determination of the aroma profile using a trained panel, the formulation of the new aroma components should be qualitatively and quantitatively analyzed. Based on the results of SDS-PAGE, the microorganisms mainly decomposed polypeptides with low and medium molecular weight. As the protein integrity provides a first indication of the allergenic potential, in vitro and in vivo studies should be performed to investigate the allergenicity of these fermented lupin proteins. In addition, it is necessary to validate the allergen structure of LPI and fermented LPI and to develop more reliable methods for the quantification of allergens. The use of fermented plant proteins in food products such as meat and dairy alternatives must be examined in practice by subsequent application trails.

## Figures and Tables

**Table 1:** Bacteria Strains.

Bacteria Strain	
*Lactobacillus reuteri*	DSM 20016
*Lactobacillus brevis*	TMW 1.1326
*Lactobacillus amylolyticus*	TL 5
*Lactobacillus parabuchneri*	DSM 5987
*Lactobacillus sakei* subsp. *carnosus*	DSM 15831
*Staphylococcus xylosus*	DSM 20266
*Lactobacillus helveticus*	DSM 20075
*Lactobacillus delbrueckii*	DSM 20081

**Table 2:** Dry matter and protein content of unfermented lupin protein isolate (LPI) and fermented (24 h) LPI.

	Dry Matter	Protein Content
LPI (unfermented)	95.4 ± 0.0%	89.6 ± 0.0%
*L. reuteri*	94.6 ± 0.0% *	82.4 ± 1.3%
*L. brevis*	94.9 ± 0.9%	80.1 ± 0.5% *
*L. amylolyticus*	94.7 ± 0.2%	79.7 ±0.7% *
*L. parabuchneri*	94.7 ± 0.0% *	81.7 ± 0.2% *
*L. sakei* subsp. *carnosus*	94.2 ± 0.4%	80.6 ± 1.5%
*S. xylosus*	95.3 ± 1.1%	80.1 ± 1.3%
*L. helveticus*	94.7 ± 0.2%	78.5 ± 0.4% *
*L. delbrueckii*	92.8 ± 1.4%	78.5 ± 1.7%

The data are expressed as mean ± standard deviation (*n* = 4). Means marked with an asterisk (*) within a column indicate significant differences between the individual sample and the unfermented LPI (*p* < 0.05) following pairwise *t*-test.

**Table 3:** Colony forming units (CFU) (**a**) and pH, and glucose amount (**b**) after 0 h, 4 h, 18 h and 24 h of fermentation.

(**a**)									
	**CFU (CFU/mL)**								
	**0 h**		**4 h**		**18 h**		**24 h**		**ΔE_CFU_^1^**
*L. reuteri*	2.68 × 10^6^ ± 2.40 × 10^5 a^		6.52 × 10^6^ ± 2.02 × 10^6 a^		5.77 × 10^7^ ± 5.16 × 10^6 a^		1.63 × 10^7^ ± 2.83 × 10^6 a^		1.36 × 10^7^ ± 3.07 × 10^6 a^
*L. brevis*	1.83 × 10^7^ ± 2.33 × 10^6 a^		4.20 × 10^7^ ± 8.91 × 10^6 a^		1.24 × 10^8^ ± 3.75 × 10^7 a^		1.31 × 10^8^ ± 1.16 × 10^8 a^		1.13 × 10^8^ ± 1.19 × 10^8 a^
*L. amylolyticus*	1.38 × 10^7^ ± 7.38 × 10^6 a^		4.39 × 10^6^ ± 1.11 × 10^6 a^		5.77 × 10^7^ ± 2.33 × 10^6 a^		5.88 × 10^7^ ± 4.60 × 10^6 a^		4.50 × 10^7^ ± 1.20 × 10^7 a^
*L. parabuchneri*	1.21 × 10^7^ ± 3.39 × 10^6 a^		2.30 × 10^7^ ± 1.00 × 10^7 a^		3.43 × 10^7^ ± 7.07 × 10^5 a^		5.91 × 10^7^ ± 4.62 × 10^7 a^		4.70 × 10^7^ ± 4.29 × 10^7 a^
*L. sakei* subsp. *carnosus*	1.95 × 10^7^ ± 9.19 × 10^6 a^		1.74 × 10^7^ ± 5.09 × 10^6 a^		1.33 × 10^7^ ± 3.32 × 10^6 a^		3.99 × 10^7^ ± 1.56 × 10^6 a^		2.04 × 10^7^ ± 7.64 × 10^6 a^
*S. xylosus*	1.24 × 10^7^ ± 1.54 × 10^7 a^		3.13 × 10^7^ ± 1.27 × 10^7 a^		1.86 × 10^8^ ± 1.91 × 10^8 a^		6.13 × 10^8^ ± 7.59 × 10^8 a^		6.01 × 10^8^ ± 7.75 × 10^8 a^
*L. helveticus*	3.08 × 10^7^ ± 1.25 × 10^7 a^		3.67 × 10^7^ ± 2.03 × 10^7 a^		5.09 × 10^7^ ± 1.12 × 10^7 a^		7.44 × 10^7^ ± 1.94 × 10^7 a^		4.37 × 10^7^ ± 3.19 × 10^7 a^
*L. delbrueckii*	3.10 × 10^6^ ± 9.97 × 10^5 a^		3.01 × 10^6^ ± 1.77 × 10^5 a^		4.34 × 10^7^ ± 1.51 × 10^7 a^		1.20 × 10^8^ ± 1.84 × 10^7 a^		1.17 × 10^8^ ± 1.74 × 10^7 a^
(**b**)									
	**pH**				**Glucose (g/kg)**				
	**0 h**	**4 h**	**18 h**	**24 h**	**0 h**	**4 h**	**18 h**	**24 h**	**ΔE_Glucose_^2^**
*L. reuteri*	6.5 ± 0.0 ^b^	6.4 ± 0.0 ^c,d,e^	4.9 ± 0.0 ^b,c,d^	4.8 ± 0.0 ^b,c^	5.0 ± 0.0 ^a^	4.1 ± 0.2 ^a,b,c^	0.9 ± 0.1 ^a^	0.2 ± 0.2 ^a^	4.8 ± 0.0 ^d^
*L. brevis*	6.6 ± 0.0 ^b,c^	6.5 ± 0.1 ^d,e^	5.3 ± 0.4 ^c,d,e^	5.0 ± 0.3 ^b,c^	5.0 ± 0.0 ^a^	4.7 ± 0.3 ^d^	3.7 ± 1.1 ^e^	0.9 ± 0.3 ^d^	4.1 ± 0.2 ^b^
*L. amylolyticus*	6.6 ± 0.0 ^c^	6.2 ± 0.0 ^c^	5.6 ± 0.3 ^e^	5.2 ± 0.1 ^c^	5.0 ± 0.0 ^a^	4.1 ± 0.1 ^a,b,c^	2.5 ± 0.0 ^c,d^	1.7 ± 0.3 ^e^	3.4 ± 0.1 ^a^
*L. parabuchneri*	6.6 ± 0.0 ^c^	6.5 ± 0.0 ^d,e^	5.5 ± 0.0 ^d,e^	4.9 ± 0.0 ^b,c^	5.0 ± 0.0 ^a^	4.3 ± 0.2 ^b,c,d^	3.3 ± 0.3 ^d,e^	0.7 ± 0.1 ^c,d^	4.3 ± 0.1 ^b,c^
*L. sakei* subsp. *carnosus*	6.6 ± 0.0 ^c^	6.3 ± 0.0 ^c,d^	4.7 ± 0.0 ^a,b,c^	4.7 ± 0.0 ^b^	5.0 ± 0.0 ^a^	4.4 ± 0.4 ^c,d^	1.4 ± 0.0 ^a,b^	0.4 ± 0.1 ^a,b^	4.6 ± 0.1 ^c,d^
*S. xylosus*	6.6 ± 0.0 ^c^	6.5 ± 0.0 ^e^	4.8 ± 0.1 ^b,c^	4.8 ± 0.0 ^b,c^	5.0 ± 0.0 ^a^	4.3 ± 0.2 ^b,c,d^	1.4 ± 0.3 ^a,b^	0.5 ± 0.4 ^a,b,c^	4.5 ± 0.1 ^c,d^
*L. helveticus*	6.0 ± 0.0 ^a^	5.4 ± 0.1 ^a^	4.1 ± 0.2 ^a^	3.9 ± 0.2 ^a^	5.0 ± 0.0 ^a^	3.8 ± 0.1 ^a^	1.8 ± 0.2 ^b^	0.6 ± 0.1 ^b,c,d^	4.4 ± 0.0 ^b,c^
*L. delbrueckii*	6.0 ± 0.1 ^a^	6.0 ± 0.0 ^b^	4.2 ± 0.1 ^a,b^	4.4 ± 0.0 ^a^	5.0 ± 0.0 ^a^	4.0 ± 0.4 ^a,b^	2.1 ± 0.2 ^b,c^	0.8 ± 0.2 ^c,d^	4.2 ± 0.1 ^b,c^

The data are expressed as mean ± standard deviation from duplicates. Values followed by different letter in a column indicate significant differences between groups (*p* < 0.05) following one-way ANOVA (Tukey). ^1^ ΔE_CFU_ = Growth rate over 24 h fermentation; ^2^ ΔE_Glucose_ = Difference of the start and end glucose content of the fermentation.

**Table 4:** Protein solubility (%) at pH 4 and pH 7 of unfermented und fermented (24 h) LPI.

Samples	Protein Solubility	
	pH 4	pH 7
LPI (unfermented)	7.31 ± 0.26	63.59 ± 3.04
*L. reuteri*	7.40 ± 0.69	42.35 ± 3.76 *
*L. brevis*	7.41 ± 0.97	38.45 ± 2.87 *
*L. amylolyticus*	7.13 ± 0.31	35.47 ± 3.16 *
*L. parabuchneri*	8.01 ± 0.90	27.37 ± 4.00 *
*L. sakei* subsp. *carnosus*	7.17 ± 1.01	37.40 ± 4.53 *
*S. xylosus*	8.11 ± 0.77 *	28.04 ± 3.01 *
*L. helveticus*	5.92 ± 0.92 *	23.57 ± 2.99 *
*L. delbrueckii*	5.55 ± 0.41 *	27.48 ± 2.51 *

The data are expressed as mean ± standard deviation (*n* = 4). Means marked with an asterisk (*) within a column indicate significant differences between sample and unfermented LPI (*p* < 0.05) following pairwise *t*-test.

**Table 5:** Foam activity (%) and foam stability (%) of unfermented and fermented (24 h) LPI.

Samples	Foam Activity (%)	Foam Stability (%)
LPI (unfermented)	1613 ± 11	89 ± 3
*L. reuteri*	1646 ± 20 *	87 ± 3
*L. brevis*	1683 ± 57	86 ± 6
*L. amylolyticus*	1688 ± 52 *	94 ± 2
*L. parabuchneri*	1703 ± 25 *	16 ± 5 *
*L. sakei* subsp. *carnosus*	1670 ± 32 *	83 ± 3
*S. xylosus*	1678 ± 23 *	91 ± 1
*L. helveticus*	1698 ± 17 *	20 ± 0 *
*L. delbrueckii*	1652 ± 36	80 ± 0 *

The data are expressed as mean ± standard deviation (*n* = 4). Means marked with an asterisk (*) within a column indicate significant differences between sample and unfermented LPI (*p* < 0.05) following pairwise *t*-test.

**Table 6:** Emulsifying capacity (mg/mL) of unfermented and fermented (24 h) LPI.

Samples	Emulsifying Capacity (mg/mL)
LPI (unfermented)	552.9 ± 9.8
*L. reuteri*	471.5 ± 10.7 *
*L. brevis*	447.2 ± 27.4 *
*L. amylolyticus*	564.1 ± 18.7
*L. parabuchneri*	347.7 ± 5.2 *
*L. sakei* subsp. *carnosus*	407.2 ± 7.6 *
*S. xylosus*	595.6 ± 55.6
*L. helveticus*	455.2 ± 51.9
*L. delbrueckii*	370.3 ± 6.8 *

The data are expressed as mean ± standard deviation (*n* = 4). Values marked with an asterisk (*) indicate significant differences between the individual sample and the unfermented LPI (*p* < 0.05) following pairwise *t*-test.
